# Micro-bursts matter: vigorous intermittent lifestyle physical activity and risk of common brain disorders in a prospective cohort study

**DOI:** 10.1186/s12966-026-01966-1

**Published:** 2026-08-18

**Authors:** Chang Liu, Jiang-Yan Wang, Xiao-Yu Jia, Cun-Xian Jia, Bao-Peng Liu

**Affiliations:** Department of Epidemiology, School of Public Health, Cheeloo College of Medicine, Shandong University, Jinan, Shandong China

**Keywords:** Accelerometer, Brain disorders, Dose-response relationship, Physical activity, Prospective cohort

## Abstract

**Background:**

Physical activity, particularly moderate-to-vigorous physical activity, is important for brain health; however, many adults do not meet recommended activity levels. Vigorous intermittent lifestyle physical activity (VILPA) may provide a feasible alternative. This study examined the associations of accelerometer-derived vigorous intermittent lifestyle physical activity with the risk of five common brain disorders.

**Methods:**

This prospective cohort study used accelerometer data from the UK Biobank to quantify daily VILPA according to both duration and standardized frequency, using bouts lasting up to 1 min and up to 2 min. Incident anxiety, depression, dementia, Parkinson’s disease, and stroke were ascertained through linked health records. Cox proportional hazards models were used to estimate hazard ratios (HRs) and 95% confidence intervals (CIs). Restricted cubic spline models were applied to examine dose-response associations. Analyses were further stratified by exercise habit status.

**Results:**

During a median follow-up of approximately 8 years, 2,150 anxiety (2.47%), 1,511 depression (1.81%), 676 dementia (0.74%), 343 Parkinson’s disease (0.37%), and 1,385 stroke cases (1.55%) were identified. Higher VILPA frequency and duration were associated with lower risks of several brain disorders. Compared with the lowest quartile, modest VILPA levels were associated with reduced risks of anxiety (HR = 0.80, 95% CI: 0.71–0.90), depression (HR = 0.86, 95% CI: 0.75–0.99), dementia (HR = 0.72, 95% CI: 0.59–0.88), and Parkinson’s disease (HR = 0.58, 95% CI: 0.43–0.78). Associations with stroke were weaker and less consistent. Dose-response analyses showed inverse nonlinear associations for anxiety, depression, dementia, and Parkinson’s disease, characterized by a steep initial decline followed by a gradual plateau. Similar patterns were observed among both exercisers and non-exercisers.

**Conclusions:**

VILPA was associated with lower risks of several common brain disorders. Incorporating brief bursts of vigorous movement into daily life may represent a feasible strategy for promoting brain health.

**Supplementary Information:**

The online version contains supplementary material available at https://doi.org/10.1186/s12966-026-01966-1.

## Background

The global burden of brain disorders, including neurological and psychiatric conditions, represents a major public health challenge in the 21st century [1, 2]. These disorders substantially impair quality of life and place considerable pressure on healthcare systems worldwide [3, 4]. Physical activity is widely regarded as an important correlate of brain health, and evidence from systematic reviews and meta-analyses suggests that higher levels of physical activity are associated with lower risks of several brain disorders, including anxiety [5], depression [6], dementia [7], Parkinson’s disease [8], and stroke [9]. In particular, moderate-to-vigorous physical activity (MVPA) has been associated with better cognitive function and a lower risk of dementia and Parkinson’s disease [6–10]. The World Health Organization recommends at least 150 min of moderate physical activity (MPA) or 75 min of vigorous physical activity (VPA) per week [11]. 

Despite these recommendations, physical activity levels remain insufficient among many adults [12, 13]. Studies indicate that nearly one-third of adults worldwide fail to meet the physical activity guidelines [13]. This highlights the challenges of promoting continuous, structured exercise, particularly for individuals facing barriers such as time constraints. Moreover, much of the existing evidence on physical activity and brain health relies on self-reported questionnaires [14], which typically focus on larger blocks of activity over extended periods, often overlooking shorter bursts of activity that occur throughout the day. By contrast, accelerometers provide objective, high-resolution measurements of physical activity and can detect short periods of movement that are not easily captured by self-report [15–17]. 

Vigorous intermittent lifestyle physical activity (VILPA) is a promising activity pattern in this context. It refers to brief, intense bursts of physical activity, usually lasting up to 1–2 min, that occur as part of daily life rather than structured exercise [18, 19]. Unlike VPA, VILPA is more accessible for individuals who may find it difficult to commit to longer exercise sessions. Previous studies have demonstrated that VILPA has been associated with lower risks of all-cause mortality, cancer incidence and mortality, as well as cardiovascular disease incidence and mortality [19–21]. Although recent reviews and systematic syntheses have increasingly examined incidental physical activity and short or accumulated bouts of activity [22, 23], evidence specifically relating these brief activity patterns to common brain disorders remains relatively limited.

In summary, this study aims to investigate the associations between VILPA and the risk of five common brain disorders, namely anxiety, depression, dementia, Parkinson’s disease, and stroke using data from the UK Biobank. By analyzing the frequency and duration of VILPA, we sought to determine whether VILPA was associated with a lower risk of brain disorders and whether such associations were present in both exercisers and non-exercisers.

## Methods

### Study design and participants

The UK Biobank (UKB) is a large, ongoing prospective cohort study. More than 500,000 participants, aged 37 to 73 years at baseline, were enrolled between 2006 and 2010 across 22 centers in the UK [24]. Participants provided written informed consent, completed touchscreen questionnaires, underwent physical examinations, and provided biological samples [25]. From 2013 to 2015, a random subset of 236,519 participants with a valid email address was invited to wear a wrist accelerometer for 7 consecutive days. A total of 106,053 individuals consented to wear the Axivity AX3 triaxial device (Axivity Ltd, Newcastle, UK) [26]. 

**Fig. 1:**
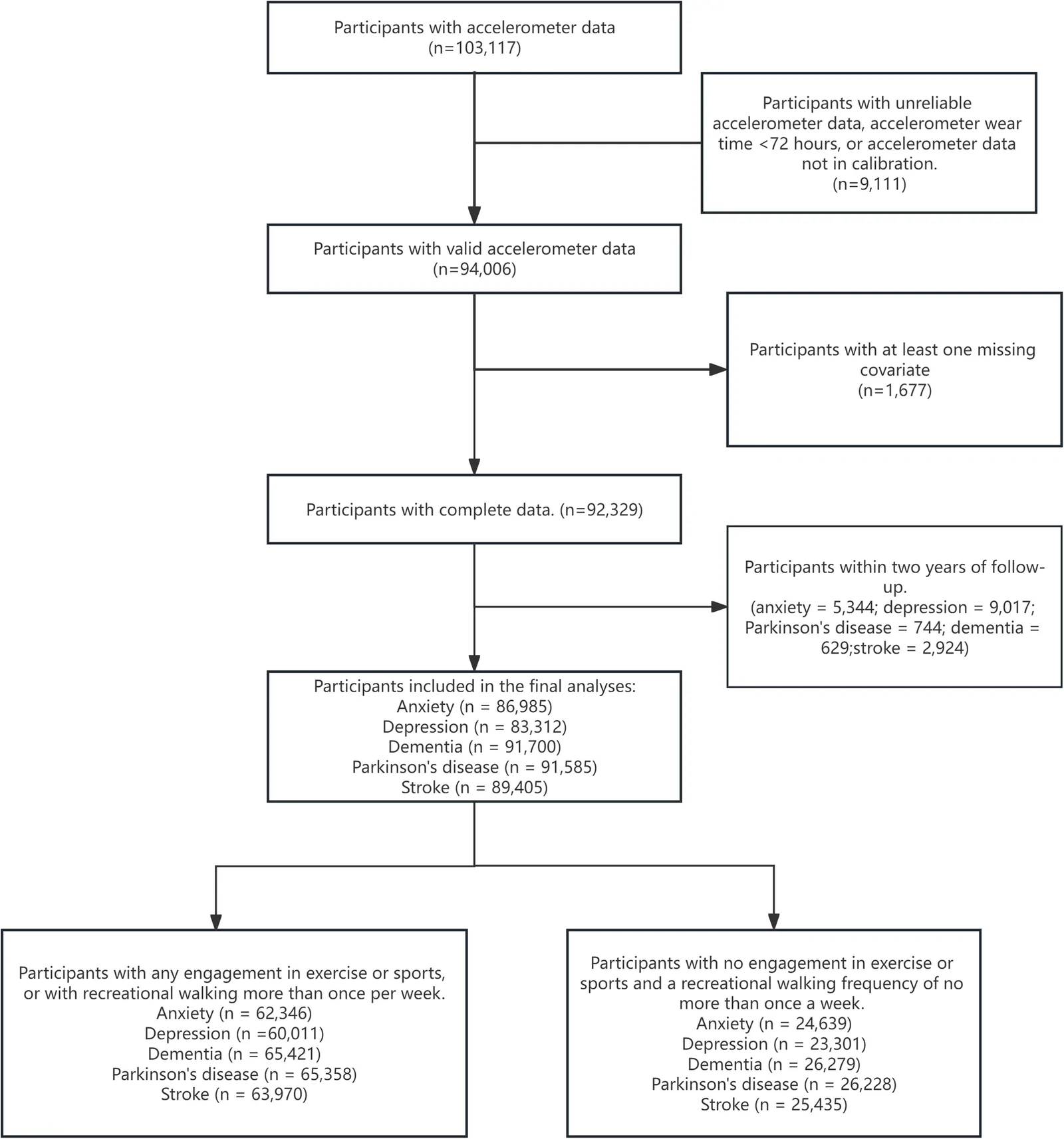
Flow diagram of UK Biobank participants for the VILPA

Participants were first excluded on the basis of accelerometer data quality, including implausibly high or low values identified by the UKB Accelerometer Expert Group (*n* = 4,664), fewer than 72 h of wear time, at least 1 h of missing recording within any 24-hour period during the 7-day monitoring period (*n* = 4,444), or uncalibrated signals (*n* = 3). Participants with a diagnosis of the corresponding brain disorder before accelerometer assessment were then excluded. Follow-up began two years after the accelerometer wear date, and events occurring within the first two years after assessment were also excluded [19, 25]. Participants with missing covariate information were additionally excluded. The final analytic samples included 86,985 participants for anxiety, 83,312 for depression, 91,700 for dementia, 91,585 for Parkinson’s disease, and 89,405 for stroke. The participants’ selection process is shown in Fig. 1.

### Exposure

Physical activity intensity was classified according to established accelerometer thresholds, with light physical activity (LPA) defined as 30 to 125 mg (milli-gravitational units), MPA ranging from greater than 125 to 400 mg, and VPA including all values exceeding 400 mg [27]. VILPA was defined as the subset of VPA bouts lasting up to 1 min or up to 2 min. These time thresholds were selected based on previous studies, which reported that, across different kinds of physical activities, the average time required to reach vigorous intensity was 73.5 (26.2) seconds [28]. 

Accelerometer data processed in 5-second epochs by the UK Biobank Accelerometer Expert Group were used. The average acceleration value for each 5-second epoch was used to classify physical activity. Continuous 5-second epochs with acceleration values exceeding 400 mg were aggregated to identify VILPA. If the VPA bout lasted up to 1/2 min, it was considered a VILPA episode. Following the approach described by Stamatakis et al. (2022), frequency was derived from accelerometer time-series data using a rolling-sum approach to standardize raw activity into bouts of 1 min and 2 min [19]. This procedure allowed VILPA frequency to be compared across participants without being disproportionately influenced by differences in individual bout duration.

We also stratified participants by exercise habit status. In line with the definition proposed by Caspersen et al., exercise was considered a planned, structured, and intentional form of physical activity performed to maintain or improve physical fitness [29]. Participants were classified as non-exercisers if they reported no participation in leisure-time exercise and reported leisure walking no more than once per week. Conversely, participants were classified as exercisers if they reported participation in leisure-time exercise or leisure walking more than once per week. Leisure-time exercise and walking were assessed using closed-ended touchscreen questionnaires on participation, frequency, and, where applicable, duration (Supplementary Table 3).

### Covariates

Covariates were selected a priori based on previous literature and prior knowledge [27, 30]. Covariates were age, sex, ethnicity, socioeconomic status (SES), body mass index (BMI), household income before tax per year, educational attainment, smoking status, and alcohol drinking status. SES was assessed using the Townsend area deprivation index, with higher scores indicating greater material deprivation and thus lower SES [31, 32]. In addition, models were further adjusted for time spent in LPA and MPA, as well as the duration of bouts above the specified bout threshold within each frequency/duration analysis [19, 20]. Detailed definitions of all covariates are presented in Supplementary Table 1.

### Outcome

Drawing on prior research on brain disorders and considering that these conditions contribute substantially to global disease burden and are especially prevalent in older adults, we examined five common conditions: dementia, stroke, Parkinson’s disease, depression, and anxiety [33, 34]. Outcomes were identified and classified according to the corresponding International Classification of Diseases codes (Supplementary Table 2). Ascertainment relied on the UK Biobank “first occurrence” data fields, which compile information from hospital inpatient records, self-reported medical conditions, primary care records, and national death registry data. Participants were censored at the date of first diagnosis of the corresponding outcome, death, or the end of available follow-up, whichever occurred first. The censoring date was 31 October 2022 for England, 31 August 2022 for Scotland, and 31 May 2022 for Wales. Dates and causes of death were obtained from the National Health Service Information Centre and the NHS Central Register death registries.

### Statistical analysis

Characteristics were presented as means±standard deviations (SD) for normally distributed continuous variables and frequencies (percentages) for categorical variables. The durations of LPA and MPA were expressed as medians and interquartile ranges. VILPA duration and standardized frequency, each derived using bout definitions of up to 1 min and up to 2 min, were categorized into quartiles, with the lowest quartile (Q1) serving as the reference group. Cox proportional hazards regression models were used to estimate hazard ratios (HRs) and 95% confidence intervals (CIs) for incident brain disorders. Model 1 was an unadjusted Cox model. Model 2 was adjusted for a consistent set of covariates.

The cumulative incidence of brain disorders across VILPA quartiles was estimated using Kaplan-Meier curves and compared via log-rank tests. To further characterize the dose-response relationships, we employed restricted cubic splines in the Cox models using the continuous forms of daily VILPA duration and standardized VILPA frequency based on bouts lasting up to 1 min and up to 2 min. Restricted cubic splines were specified with four knots placed at the default percentile locations of each exposure distribution. Furthermore, stratified and moderation analyses were performed according to exercise status, with non-linearity and interaction assessed using Wald tests.

Sensitivity analyses were also conducted. First, we calculated E-values for the main effect estimates to quantify the minimum strength of association. The E-value represents the minimum strength of association that an unmeasured confounder would need to have with both VILPA and the outcome, conditional on the measured covariates, to fully explain away the observed association. Second, we performed additional adjustment analyses by separately adding cognitive function, prevalent cardiovascular disease, and self-rated health status to the fully adjusted models. Cognitive function included visual memory and reaction time [35]. All statistical analyses were conducted using R (version 4.4.1; R Foundation for Statistical Computing), with statistical significance defined as a two-sided *P* < 0.05.

## Results

### Characteristics of participants

Table 1 presents the characteristics of the 86,985 participants included in the anxiety outcome analysis. During a median follow-up of 7.96 years, 2,150 participants developed incident anxiety, corresponding to an incidence proportion of 2.47%. Among all identified VPA bouts, 77.6% lasted up to 1 min and 82.7% lasted up to 2 min. Participants had a mean age of 62.32 (SD: 7.85) years, with 55.9% females. The majority of participants were White (96.9%), and 60.4% were classified as overweight or obese. For VILPA frequency groups, participants were divided into quartiles with Q1 as ≤ 1.12, Q2 as 1.12 to 2.32, Q3 as 2.32 to 4.40, and Q4 as > 4.40 standardized bouts per day. Additionally, the incidence proportions were 1.81% (1,511/83,312) for depression, 0.74% (676/91,700) for dementia, 0.37% (343/91,585) for Parkinson’s disease, and 1.55% (1,385/89,405) for stroke. In the linked death registry data, no deaths were attributed to anxiety or depressive disorders, whereas 96, 21, and 112 deaths were attributed to dementia, Parkinson’s disease, and stroke, respectively. The distribution of baseline characteristics showed similar patterns across the various outcome analysis samples. The baseline characteristics of participants included in the remaining outcome-specific analyses are provided in Supplementary Tables 4–7.

**Table 1:** Characteristics of participants included in the anxiety outcome analysis (*n* = 86, 985) by VILPA standardized frequency

	Overall	Daily VILPA standardized frequency (lasting up to 2 min)			
		≤ 1.12	>1.12–2.32	>2.32–4.40	> 4.40
*n*	86,985	21,747	21,746	21,746	21,746
Age, mean (SD)	62.32 (7.85)	65.24 (7.20)	62.86 (7.68)	61.49 (7.74)	59.67 (7.69)
Sex, *n* (%)					
Female	48,611 (55.9)	13,741 (63.2)	12,563 (57.8)	11,625 (53.5)	10,682 (49.1)
Male	38,374 (44.1)	8006 (36.8)	9183 (42.2)	10,121 (46.5)	11,064 (50.9)
Ethnicity, *n* (%)					
White	84,284 (96.9)	21,170 (97.3)	21,121 (97.1)	21,086 (97.0)	20,907 (96.1)
Other	2701 (3.1)	577 (2.7)	625 (2.9)	660 (3.0)	839 (3.9)
Socioeconomic status^b^, *n* (%)					
First quartile	24,891 (28.6)	5800 (26.7)	6212 (28.6)	6463 (29.7)	6416 (29.5)
Second quartile	23,152 (26.6)	5624 (25.9)	5699 (26.2)	5909 (27.2)	5920 (27.2)
Third quartile	21,808 (25.1)	5518 (25.4)	5459 (25.1)	5341 (24.6)	5490 (25.2)
Fourth quartile	17,134 (19.7)	4805 (22.1)	4376 (20.1)	4033 (18.5)	3920 (18.0)
BMI, n (%)					
Underweight or normal weight (< 24.9 kg/m2)	34,451 (39.6)	6484 (29.8)	7975 (36.7)	9201 (42.3)	10,791 (49.6)
Overweight (25.0–29.9 kg/m2)	35,854 (41.2)	8891 (40.9)	9230 (42.4)	9090 (41.8)	8643 (39.7)
Obese (≥ 30 kg/m2)	16,680 (19.2)	6372 (29.3)	4541 (20.9)	3455 (15.9)	2312 (10.6)
Household income before tax per year, (£), *n* (%)					
Less than 18, 000	11,081 (12.7)	3724 (17.1)	2812 (12.9)	2390 (11.0)	2155 (9.9)
18, 000–30, 999	18,720 (21.5)	5317 (24.4)	4826 (22.2)	4484 (20.6)	4093 (18.8)
31, 000–51, 999	22,584 (26.0)	5191 (23.9)	5633 (25.9)	5840 (26.9)	5920 (27.2)
Greater than 52, 000	25,852 (29.7)	5024 (23.1)	6193 (28.5)	7003 (32.2)	7632 (35.1)
Unknown^a^	8748 (10.1)	2491 (11.5)	2282 (10.5)	2029 (9.3)	1946 (8.9)
Educational group, *n* (%)					
College or university degree	37,769 (43.4)	9151 (42.1)	9324 (42.9)	9499 (43.7)	9795 (45.0)
Any school degree (A-level, AS-level, O-level, GCSE, CSE)	32,416 (37.3)	7789 (35.8)	8084 (37.2)	8213 (37.8)	8330 (38.3)
Vocational qualification (NVQ, HND, or HNC) or other professional qualifications	8996 (10.3)	2439 (11.2)	2263 (10.4)	2242 (10.3)	2052 (9.4)
None of the above	7004 (8.1)	2117 (9.7)	1874 (8.6)	1607 (7.4)	1406 (6.5)
Unknown	800 (0.9)	251 (1.2)	201 (0.9)	185 (0.9)	163 (0.7)
Smoking status, *n* (%)					
Smoker	5799 (6.7)	1732 (8.0)	1462 (6.7)	1381 (6.4)	1224 (5.6)
Non-smoker	81,186 (93.3)	20,015 (92.0)	20,284 (93.3)	20,365 (93.6)	20,522 (94.4)
Alcohol drinking status, *n* (%)					
Non-moderate drinking	28,938 (33.3)	6416 (29.5)	7105 (32.7)	7602 (35.0)	7815 (35.9)
Moderate drinking	58,047 (66.7)	15,331 (70.5)	14,641 (67.3)	14,144 (65.0)	13,931 (64.1)
Non-exercisers, *n* (%)	24,639 (28.3)	8100 (37.2)	6671 (30.7)	5644 (26.0)	4224 (19.4)
Exercisers, *n* (%)	62,346 (71.7)	13,647 (62.8)	15,075 (69.3)	16,102 (74.0)	17,522 (80.6)
Light activity (min per day), median [IQR]	298.34 [255.67, 343.01]	274.42 [230.07, 322.20]	295.31 [254.51, 340.87]	306.79 [266.02, 349.62]	313.02 [274.04, 354.09]
Moderate activity (min per day), median [IQR]	62.10 [43.10, 84.93]	36.30 [25.05, 49.82]	55.26 [42.68, 69.99]	70.13 [55.56, 87.28]	91.81 [73.06, 114.56]

*BMI* body mass index, *IQR *interquartile range, *SD *standard deviation, *VILPA *vigorous intermittent lifestyle physical activity

^a^ Unknown included prefer not to answer, do not know, and missing value in the database of UK Biobank

^b^ Socioeconomic status was measured by Townsend area deprivation index

### Associations of VILPA with brain disorders

**Fig. 2:**
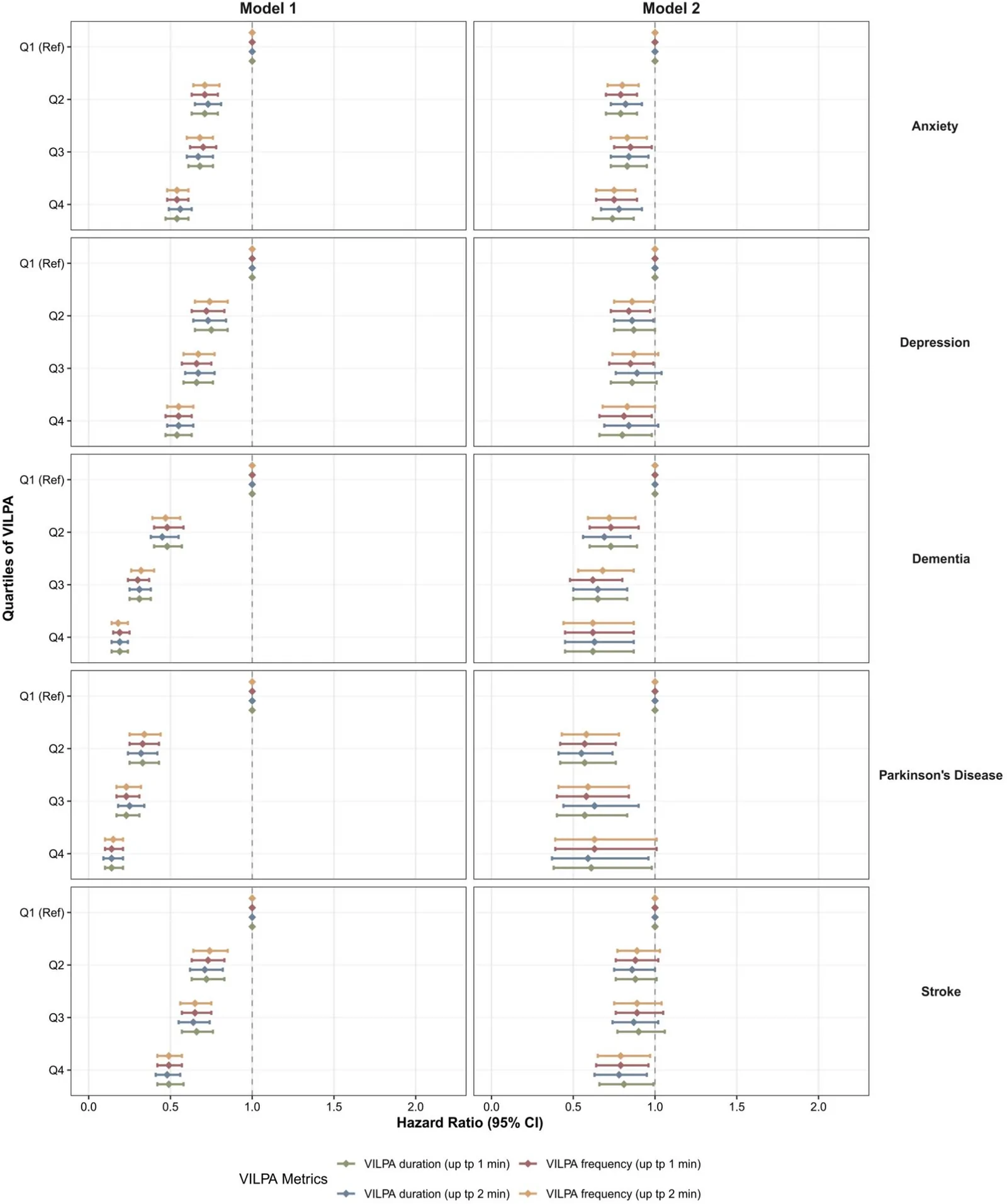
Association of the daily duration and frequency of VILPA with brain disorders. VILPA: vigorous intermittent lifestyle physical activity. Model 1 was not adjusted for any covariate. Model 2 was adjusted for age, sex, ethnicity, socioeconomic status, BMI, household income before tax per year, educational group, smoking status, alcohol drinking status, duration of light physical activity, duration of moderate physical activity, exercise habit status and vigorous physical activity lasting more than one/two minutes

Based on the results from the multivariable-adjusted model, and as shown in Fig. 2, higher VILPA frequency and duration (both 1 min and 2 min) were associated with a reduced risk of anxiety, depression, dementia, and Parkinson’s disease. The associations were relatively consistent for anxiety and dementia, whereas the estimates for Parkinson’s disease were also substantial but had wider confidence intervals. In contrast, the association with stroke was weaker and less consistent. Even in Q2, a modest increase in VILPA frequency (2 min) was associated with a significant reduction in the risk of anxiety (HR = 0.80, 95% CI: 0.71–0.90) and dementia (HR = 0.72, 95% CI: 0.59–0.88). A similar pattern was seen for depression, with a significant but weaker reduction in Q2 (HR = 0.86, 95% CI: 0.75–0.99). For Parkinson’s disease, significant risk reductions were observed in both Q2 and Q3, with reductions of 42% in Q2 (HR = 0.58, 95% CI: 0.43–0.78) and 41% in Q3 (HR = 0.59, 95% CI: 0.41–0.84). However, the reduction in Q4 did not reach statistical significance. For stroke, the highest quartile was associated with a lower risk (HR = 0.79, 95% CI: 0.64–0.96), although the association was weaker than those observed for other outcomes.

**Fig. 3:**
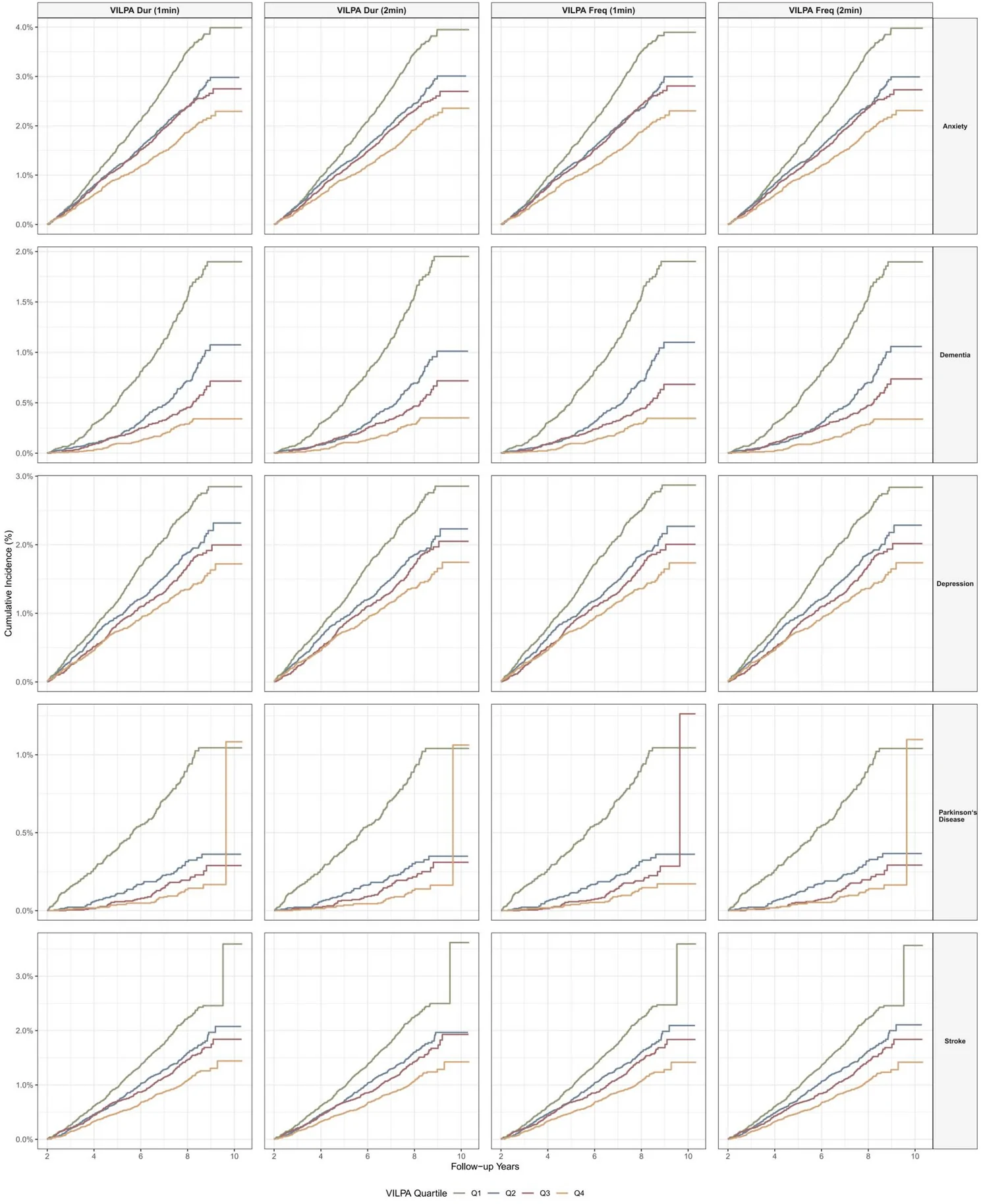
Cumulative risk of brain disorders stratified by VILPA. VILPA: vigorous intermittent lifestyle physical activity. The abrupt increases near the end of follow-up in the Parkinson’s disease and stroke curves should be interpreted cautiously, as very few participants remained at risk in some quartile groups and a single late event could produce a visibly large step increase in the cumulative incidence estimate

Figure 3 presents the cumulative risk of five brain disorders, with participants stratified into quartiles based on VILPA frequency and duration (both 1 min and 2 min). While the cumulative incidence for each of the five conditions consistently increased over the follow-up period, the rate of accrual varied significantly across the quartiles of each of the four VILPA metrics. Taking anxiety as a representative case, the cumulative incidence at a median follow-up of 7.96 years was 3.49% in the lowest quartile (Q1), compared with 1.87% in the highest quartile (Q4). This trend was consistently observed across the other four outcomes. These differences were statistically significant according to log-rank tests (*P* < 0.001) (Supplementary Table 8).

The dose-response analyses shown in Supplementary Fig. 1 were broadly consistent with these findings. Overall, VILPA was inversely associated with the risk of anxiety, depression, dementia, and Parkinson’s disease, exhibiting a nonlinear pattern characterized by a steep initial decline in risk followed by a gradual plateau. For example, for anxiety, the association with VILPA frequency (1 min) showed a rapid decline in risk between 0 and about 2 bouts per day, after which the curve gradually leveled off. In contrast, stroke demonstrated an inverse linear dose-response relationship (*P* for non-linear = 0.143).

### Associations of VILPA with brain disorders in non-exercisers and exercisers

**Fig. 4:**
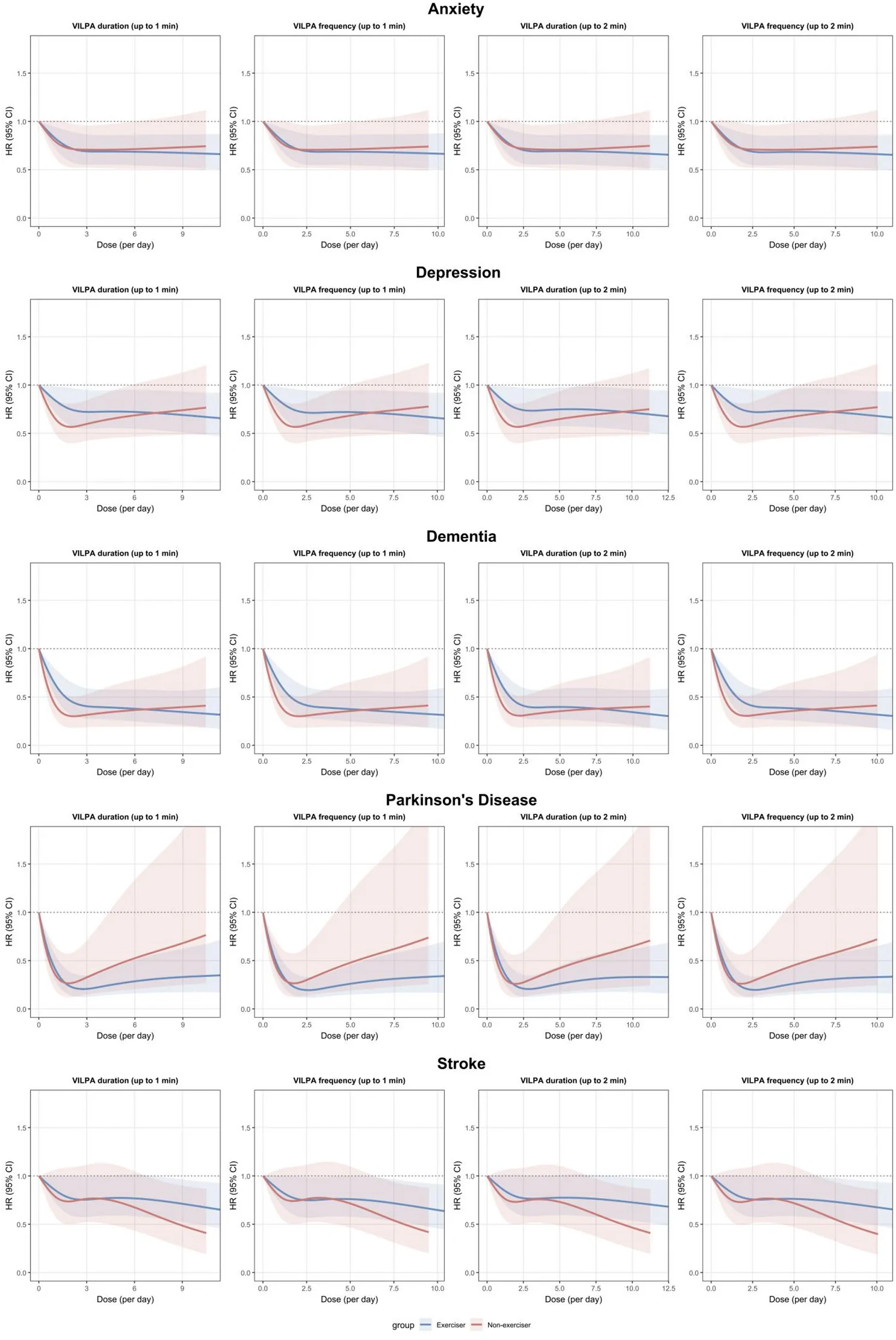
Dose-response associations between four VILPA metrics and brain disorders in non-exercisers and exercisers. VILPA: vigorous intermittent lifestyle physical activity. Model was adjusted for age, sex, ethnicity, socioeconomic status, BMI, household income before tax per year, educational group, smoking status, alcohol drinking status, duration of light physical activity, duration of moderate physical activity and vigorous physical activity lasting more than one/two minutes. Shaded areas represent 95% CIs, and solid lines represent point estimates

The subgroup analysis, as illustrated in Fig. 4, further elucidates the association between VILPA and a reduced risk of brain disorders, regardless of baseline exercise habits, and moderation analyses showed no statistically significant overall interactions by exercise status (Supplementary Table 9). Among non-exercisers, depression, dementia, and Parkinson’s disease followed an inverse nonlinear dose-response trend, consistent with the patterns observed in the overall population. The most rapid risk reduction occurred at the lower end, with the protective benefits reaching a plateau as VILPA duration and frequency increased. Notably, engaging in just 2.5 min of daily VILPA (with bouts lasting up to 1 min) was associated with a substantial 67% lower hazard of dementia (HR = 0.33, 95% CI: 0.2–0.56). A similar pattern was observed among exercisers, particularly for dementia and Parkinson’s disease, whereas anxiety in this group showed a linear inverse relationship. At modest VILPA levels, risk reductions were comparable between both groups; for instance, 2.5 min of daily VILPA was associated with a 58% reduction in dementia risk among exercisers (HR = 0.42, 95% CI: 0.29–0.62). Regarding stroke, while both groups exhibited a downward trend in risk, the associations did not reach significance.

### Sensitivity analysis

The results of E-values suggested that relatively strong unmeasured confounding would be needed to fully explain the observed associations (Supplementary Table 10). Values are presented as point estimate (CI limit). For anxiety, E-values ranged from 1.62 (1.19) to 2.06 (1.57); depression, 1.51 (1.00) to 1.79 (1.17); dementia, 2.07 (1.48) to 2.61 (1.81); Parkinson’s disease, 2.56 (1.47) to 3.03 (2.02); and stroke, 1.48 (1.00) to 1.86 (1.23). Because many stroke estimates were weak or not statistically significant, these E-values should be interpreted cautiously. In addition, separately adjusting for cognitive function, prevalent cardiovascular disease, and self-rated health status did not materially change the results (Supplementary Tables 11–13).

## Discussion

In this large cohort study, we examined the associations between VILPA and the risk of five common brain disorders, including anxiety, depression, dementia, Parkinson’s disease, and stroke. Our findings indicate that higher VILPA frequency and duration are associated with a lower risk of anxiety, depression, dementia, and Parkinson’s disease. Notably, substantial risk reductions were observed even with minimal increases in VILPA. These patterns were broadly similar among both exercisers and non-exercisers. However, the association with stroke was weaker and less consistent than those observed for other brain disorders.

Our findings support and extend the current public health message that every move counts [11]. Within this broader framework, VILPA may be viewed as a specific form of lifestyle physical activity characterized by brief bursts of vigorous intensity during daily routines. This distinction may be relevant because VILPA captures short episodes of high intensity movement embedded in everyday life that might not be fully reflected by conventional activity measures focused on structured exercise or longer activity bouts [19, 21, 36]. Our results suggest that these brief vigorous bursts could represent an additional way to accumulate health-promoting activity in daily life.

Both VILPA frequency and accumulated duration were associated with lower risks of several brain disorders, particularly anxiety, dementia, and Parkinson’s disease, with more modest associations for depression. Importantly, these associations were already evident when VILPA was defined using bouts up to 1 min, suggesting that the benefits do not depend on sustained vigorous exercise sessions. Instead, repeatedly accumulating very short bursts of activity during everyday life may provide health benefits. Similarly, greater accumulated VILPA duration was associated with lower risks of anxiety, dementia, and Parkinson’s disease, with more modest associations for depression. Dose-response analyses showed that, for most outcomes, the nonlinear decline suggested that even small increases in vigorous activity may produce meaningful early benefits. A broadly similar dose-response pattern was also observed for VILPA frequency.

Our findings extend the benefits of physical activity for brain health. Previous studies have shown that VPA is associated with a reduced risk of anxiety [37, 38], depression [38], dementia [39], Parkinson’s disease [40], and stroke [41]. Extending this line of research, our findings suggest that VILPA, as a brief and lifestyle-embedded form of vigorous activity, may also be relevant to brain health.

Differences across outcomes were also observed. For Parkinson’s disease, the associations were also substantial, although they were not entirely monotonic, which may partly reflect the relatively smaller number of incident cases and the resulting statistical uncertainty. For stroke, our findings are consistent with previous research, suggesting that the association between VILPA and stroke risk may be weaker and less consistent than those observed for other outcomes [20]. By comparison, current evidence for cerebrovascular prevention is more firmly established for regular moderate-to-vigorous physical activity, including vigorous physical activity performed in more structured or sustained patterns [9]. 

Several biological pathways may help explain these associations. VILPA may operate through pathways shared with MVPA and VPA more broadly, including neuroplasticity, inflammatory regulation, cardiometabolic health, vascular function, and cardiorespiratory fitness [42]. However, its brief, intermittent, and high-intensity pattern, characterized by repeated vigorous bursts embedded in daily routines, could plausibly elicit acute physiological stimuli resembling those induced by high-intensity interval training (HIIT). Evidence from HIIT studies suggests that such stimuli may increase circulating brain-derived neurotrophic factor and induce lactate-related neuroplastic signaling [43–45]. For dementia and Parkinson’s disease, intermittent MVPA may improve cognitive function [46] and cardiorespiratory fitness [47, 48], both of which are associated with better cognitive performance and neurological resilience. Nevertheless, direct mechanistic evidence comparing VILPA with conventional MVPA remains limited, and these pathways are best interpreted as shared MVPA-related mechanisms that may be particularly relevant to intermittent vigorous patterns.

Finally, an important finding of this study is that both exercisers and non-exercisers appeared to benefit from VILPA. For exercisers, VILPA may complement regular exercise and improve their overall activity profile. For non-exercisers, brief vigorous activities integrated into daily routines may offer a practical and achievable way to reduce risk meaningfully. These findings highlight VILPA as a feasible and inclusive strategy for promoting brain health, especially among individuals with limited time or low levels of structured physical activity.

### Strengths and limitations

Our study has several strengths. First, accelerometer data provided objective measures of physical activity intensity, reducing bias from self-report. Second, the large UK Biobank sample improved statistical power and enabled analyses across multiple outcomes. Additionally, excluding participants with follow-up of 2 years or less also helped reduce reverse causality. However, there are limitations. Although we classified physical activity intensity based on accelerometer-measured magnitudes, this method may overlook certain activities in specific contexts. In addition, because VILPA was assessed during a single 7-day accelerometer monitoring period without repeated objective measurements during follow-up, it may not fully reflect participants’ long-term activity patterns and may be subject to exposure misclassification. Nevertheless, studies have shown that it is highly consistent with the gold standard [27, 49]. Despite controlling for numerous covariates, residual confounding may still exist, though sensitivity analyses and E-values suggest unmeasured confounders are unlikely to explain the observed associations. Finally, the questionnaires used in the study were collected at a different time point from the accelerometer measurements, but participants’ physical activity status is generally stable over time [19]. 

## Conclusions

In conclusion, VILPA was found to be associated with a reduced risk of several common brain disorders. Incorporating brief bursts of vigorous movement into daily life may offer an accessible, low-cost approach to promoting brain health.

## Supplementary Information

Supplementary Material 1.

Supplementary Material 2.

## Data Availability

UK Biobank data are available upon application from https://www.ukbiobank.ac.uk/enable-your-research/apply-for-access.

## Abbreviations

BMI: Body mass index
CI: Confidence interval
HR: Hazard ratio
HIIT: High-intensity interval training
IQR: Interquartile range
LPA: Light physical activity
MPA: Moderate physical activity
MVPA: Moderate-to-vigorous physical activity
SD: Standard deviation
SES: Socioeconomic status
UKB: UK Biobank
VILPA: Vigorous intermittent lifestyle physical activity
VPA: Vigorous physical activity
