# Supplementary material for: Micro-bursts matter: vigorous intermittent lifestyle physical activity and risk of common brain disorders in a prospective cohort study
Source: Int J Behav Nutr Phys Act. 2026 Aug 18;23:87. doi: 10.1186/s12966-026-01966-1 (PMC13602585; doi:10.1186/s12966-026-01966-1)
Supplement: Supplementary file 2 [file 12966_2026_1966_MOESM2_ESM.docx]

**Supplementary Table 1:** Covariate definitions

**Supplementary Table 2:** Outcome definitions

**Supplementary Table 3:** Questions to assess participation in leisure time physical activity

**Supplementary Table 4:** Baseline characteristics of participants included in the depression outcome analysis (n=83,312) stratified by VILPA standardized frequency.

**Supplementary Table 5:** Baseline characteristics of participants included in the dementia outcome analysis (n=91, 700) stratified by VILPA standardized fr equency.

**Supplementary Table 6:** Baseline characteristics of participants included in the Parkinson’s disease outcome analysis (n=91, 585) stratified by VILPA standardized frequency.

**Supplementary Table 7:** Baseline characteristics of participants included in the stroke outcome analysis (n=89, 405) stratified by VILPA standardized frequency.

**Supplementary Table 8:** Comparison of cumulative incidence of brain diorders across VILPA frequency and duration quartiles (1min/2min) with log-rank test results.

**Supplementary Figure 1:** Dose-response associations between four VILPA metrics and brain disorders.

**Supplementary Table 9:** Moderation analyses of the associations between VILPA and brain disorders by exercise status

**Supplementary Table 10:** E-values for dementia, stroke, Parkinson’s disease, depressive disorders, and anxiety disorders

**Supplementary Table 11:** Additional adjustments for self-rated health status.

**Supplementary Table 12:** cardiovascular disease outcomes

**Supplementary Table 13:** Additional adjustments for cognitive function

**Supplementary Table 1: Covariate definitions**

| **Covariates** | **Data field** | **Type of variables** | **Notes** |
| --- | --- | --- | --- |
| Age | 34, 52, 90010 | Continuous | Age at the time of wearing accelerometer |
| Sex | 31 | Categorical (female and male) | Self-reported sex during the initial Assessment Centre visit |
| Ethnicity | 21000 | Categorical (White or others) | Self-reported ethnic background during the initial Assessment Centre visit |
| Socioeconomic status | 22189 | Categorical (quartiles) | Townsend deprivation index was calculated prior to participant joining UK Biobank based on the preceding national census output areas. Each participant is assigned a score corresponding to the output area in which their postcard is located. |
| BMI | 21001 | Categorical (underweight or normal weight, overweight or obese) | BMI value is constructed from height and weight measured during the initial Assessment Centre visit. |
| Household income before tax per year (£) | 738 | Categorical (Less than 18000,  18000–30999,  31000–51999,  Greater than 52, 000,  Unknown) | Touchscreen question "What is the average total income before tax received by your HOUSEHOLD per year?" |
| Educational group | 6138 | Categorical (college or university degree; any school degree including A-level, AS-level, O-level, GCSE, CSE; vocational qualification (NVQ, HND, or HNC) or other professional qualifications; or none of the above) | Touchscreen question "Which of the following qualifications do you have? |
| Smoking status | 20116 | Categorical (Smoker, Non-smoker, Unknown) | Directly use the variable 20116, which is a summary for many questions about smoking. |
| Alcohol drinking status | 20117, 1558, 1568, 1578, 1588, 1598, 1608, 5364, 4407, 4418, 4429, 4440, 4451, 4462 | Categorical (Non-moderate drinking, Moderate drinking, Unknown) | Alcohol consumption (0-14 units per week) or excess alcohol consumption (> 14 units per week) |
| Duration of bouts above the specified bout in each frequency/duration analysis | 90004 | Number of bouts above the specified bout in the analysis. | Derived from accelerometer data |
| Duration of light physical activity | 90004 | The time spent in 30- to 125-mg intensity activity. | Derived from accelerometer data |
| Duration of moderate physical activity | 90004 | The time spent in >125- to 400-mg activity. | Derived from accelerometer data |

**Supplementary Table 2:** Outcome definitions

| **Outcome** | **ICD-10 code** | **Field ID** |
| --- | --- | --- |
| Dementia | F00-F03, G30 | 130836, 130838, 130840, 130840, 130842, 131036 |
| Parkinson’s disease | G20 | 131022 |
| Depression | F32-F33 | 130894, 130896 |
| Anxiety | F40-F41 | 130904, 130906 |
| Stroke | G45, G46, I60-I64 | 131056, 131058, 131360, 131362, 131366, 131368 |

**Supplementary Table 3:** Questions to assess participation in leisure time physical activity

| Types of physical activity in last 4 weeks UK Biobank Field ID: 6164: | • Walking for pleasure (not as a means of transport) |
| --- | --- |
|  | • Other exercises (eg: swimming, cycling, keep fit, bowling) |
|  | • Strenuous sports |
|  | • Light DIY (eg: pruning, watering the lawn) |
|  | • Heavy DIY (eg: weeding, lawn mowing, carpentry, digging) |
|  | • None of the above |
|  | • Prefer not to answer |
| Each time you did strenuous sports, about how long did you spend doing it? (UK Biobank field ID: 1001) | • Less than 15 minutes |
|  | • between 15 and 30 minutes |
|  | • between 30 minutes and 1 hour |
|  | • between 1 hour and 1.5 hours |
|  | • between 1.5 hours and 2 hours |
|  | between 2 and 3 hours |
|  | • over 3 hours, do not know |
|  | • prefer not to answer |
| How many times in the last 4 weeks did you do strenuous sports? (UK Biobank field ID: 991) | • Once in the last 4 weeks |
|  | • 2-3 times in the last 4 weeks |
|  | • Once a week |
|  | • 2-3 times a week |
|  | • 4-5 times a week |
|  | • Every day |
|  | • Do not know |
|  | • Prefer not to answer |
| Each time you did other exercises such as swimming, cycling, keep fit, about how long did you spend doing it? (UK Biobank field ID: 3647) | • Less than 15 minutes |
|  | • between 15 and 30 minutes |
|  | • between 30 minutes and 1 hour |
|  | • between 1 hour and 1.5 hours |
|  | • between 1.5 hours and 2 hours |
|  | between 2 and 3 hours |
|  | • over 3 hours, do not know |
|  | • prefer not to answer |
| How many times in the last 4 weeks did you do other exercises such as swimming, cycling, keep fit? (UK Biobank field ID: 3637) | • Once in the last 4 weeks |
|  | • 2-3 times in the last 4 weeks |
|  | • Once a week |
|  | • 2-3 times a week |
|  | • 4-5 times a week |
|  | • Every day |
|  | • Do not know |
|  | • Prefer not to answer7 |
| How many times in the last 4 weeks did you go walking for pleasure? (UK Biobank field ID: 971) | • Once in the last 4 weeks |
|  | • 2-3 times in the last 4 weeks |
|  | • Once a week |
|  | • 2-3 times a week |
|  | • 4-5 times a week |
|  | • Every day |
|  | • Do not know |
|  | • Prefer not to answer |

**Supplementary Table 4:** Baseline characteristics of participants included in the depression outcome analysis (n=83,312) stratified by VILPA standardized frequency.

|  | Overall | Daily VILPA standardized frequency (lasting up to 2 minutes) | | | |
| --- | --- | --- | --- | --- | --- |
|  |  | ≤1.12 | 1.12-2.35 | 2.35-4.44 | >4.44 |
| n | 83312 | 20828 | 20828 | 20828 | 20828 |
| **Age, mean (SD)** | 62.38 (7.85) | 65.32 (7.18) | 62.97 (7.67) | 61.53 (7.75) | 59.72 (7.71) |
| **Sex, n (%)** |  |  |  |  |  |
| Female | 46138 (55.4) | 13061 (62.7) | 11938 (57.3) | 10999 (52.8) | 10140 (48.7) |
| Male | 37174 (44.6) | 7767 (37.3) | 8890 (42.7) | 9829 (47.2) | 10688 (51.3) |
| **Ethnicity, n (%)** |  |  |  |  |  |
| White | 80686 (96.8) | 20259 (97.3) | 20232 (97.1) | 20177 (96.9) | 20018 (96.1) |
| Other | 2626 (3.2) | 569 (2.7) | 596 (2.9) | 651 (3.1) | 810 (3.9) |
| **Socioeconomic status^b^, n (%)** |  |  |  |  |  |
| First quartile | 24040 (28.9) | 5602 (26.9) | 5997 (28.8) | 6254 (30.0) | 6187 (29.7) |
| Second quartile | 22214 (26.7) | 5405 (26.0) | 5482 (26.3) | 5659 (27.2) | 5668 (27.2) |
| Third quartile | 20905 (25.1) | 5281 (25.4) | 5243 (25.2) | 5105 (24.5) | 5276 (25.3) |
| Fourth quartile | 16153 (19.4) | 4540 (21.8) | 4106 (19.7) | 3810 (18.3) | 3697 (17.8) |
| **BMI, n (%)** |  |  |  |  |  |
| Underweight or normal weight (< 24.9kg/m2) | 33370 (40.1) | 6354 (30.5) | 7737 (37.1) | 8912 (42.8) | 10367 (49.8) |
| Overweight (25.0–29.9kg/m2) | 34454 (41.4) | 8558 (41.1) | 8855 (42.5) | 8742 (42.0) | 8299 (39.8) |
| Obese (≥30kg/m2) | 15488 (18.6) | 5916 (28.4) | 4236 (20.3) | 3174 (15.2) | 2162 (10.4) |
| **Household income before tax per year, (£), n (%)** |  |  |  |  |  |
| Less than 18, 000 | 10318 (12.4) | 3460 (16.6) | 2644 (12.7) | 2209 (10.6) | 2005 (9.6) |
| 18, 000–30, 999 | 17910 (21.5) | 5114 (24.6) | 4611 (22.1) | 4283 (20.6) | 3902 (18.7) |
| 31, 000–51, 999 | 21608 (25.9) | 4985 (23.9) | 5391 (25.9) | 5562 (26.7) | 5670 (27.2) |
| Greater than 52, 000 | 25070 (30.1) | 4894 (23.5) | 5997 (28.8) | 6807 (32.7) | 7372 (35.4) |
| Unknown^a^ | 8406 (10.1) | 2375 (11.4) | 2185 (10.5) | 1967 (9.4) | 1879 (9.0) |
| **Educational group, n (%)** |  |  |  |  |  |
| College or university degree | 36116 (43.4) | 8739 (42.0) | 8923 (42.8) | 9094 (43.7) | 9360 (44.9) |
| Any school degree (A-level, AS-level, O-level, GCSE, CSE) | 31098 (37.3) | 7490 (36.0) | 7725 (37.1) | 7883 (37.8) | 8000 (38.4) |
| Vocational qualification (NVQ, HND, or HNC) or other professional qualifications | 8611 (10.3) | 2337 (11.2) | 2172 (10.4) | 2123 (10.2) | 1979 (9.5) |
| None of the above | 6726 (8.1) | 2029 (9.7) | 1806 (8.7) | 1555 (7.5) | 1336 (6.4) |
| Unknown | 761 (0.9) | 233 (1.1) | 202 (1.0) | 173 (0.8) | 153 (0.7) |
| **Smoking status, n (%)** |  |  |  |  |  |
| Smoker | 5368 (6.4) | 1630 (7.8) | 1321 (6.3) | 1285 (6.2) | 1132 (5.4) |
| Non-smoker | 77944 (93.6) | 19198 (92.2) | 19507 (93.7) | 19543 (93.8) | 19696 (94.6) |
| **Alcohol drinking status, n (%)** |  |  |  |  |  |
| Non-moderate drinking | 27885 (33.5) | 6214 (29.8) | 6837 (32.8) | 7333 (35.2) | 7501 (36.0) |
| Moderate drinking | 55427 (66.5) | 14614 (70.2) | 13991 (67.2) | 13495 (64.8) | 13327 (64.0) |
| **Non-exercisers, n(%)** | 23301 (28.0) | 7661 (36.8) | 6301 (30.3) | 5324 (25.6) | 4015 (19.3) |
| **Exercisers, n (%)** | 60011 (72.0) | 13167 (63.2) | 14527 (69.7) | 15504 (74.4) | 16813 (80.7) |
| **Light activity (min per day), median [IQR]** | 298.69 [256.30, 343.33] | 275.40 [231.27, 323.02] | 295.90 [254.85, 341.23] | 306.91 [266.24, 349.69] | 313.27 [274.19, 353.80] |
| **Moderate activity (min per day), median [IQR]** | 62.36 [43.42, 85.20] | 36.68 [25.31, 50.19] | 55.61 [42.99, 70.39] | 70.44 [55.86, 87.52] | 91.94 [73.14, 114.60] |

**Supplementary Table 5:** Baseline characteristics of participants included in the dementia outcome analysis (n=91, 700) stratified by VILPA standardized frequency.

|  | Overall | Daily VILPA standardized frequency (lasting up to 2 minutes) | | | |
| --- | --- | --- | --- | --- | --- |
|  |  | ≤1.11 | 1.11-2.31 | 2.31-4.39 | >4.39 |
| n | 91700 | 22925 | 22925 | 22925 | 22925 |
| **Age, mean (SD)** | 62.26 (7.85) | 65.16 (7.22) | 62.82 (7.68) | 61.44 (7.73) | 59.63 (7.69) |
| **Sex, n (%)** |  |  |  |  |  |
| Female | 51797 (56.5) | 14622 (63.8) | 13390 (58.4) | 12397 (54.1) | 11388 (49.7) |
| Male | 39903 (43.5) | 8303 (36.2) | 9535 (41.6) | 10528 (45.9) | 11537 (50.3) |
| **Ethnicity, n (%)** |  |  |  |  |  |
| White | 88899 (96.9) | 22328 (97.4) | 22276 (97.2) | 22238 (97.0) | 22057 (96.2) |
| Other | 2801 (3.1) | 597 (2.6) | 649 (2.8) | 687 (3.0) | 868 (3.8) |
| **Socioeconomic status^b^, n (%)** |  |  |  |  |  |
| First quartile | 26173 (28.5) | 6082 (26.5) | 6517 (28.4) | 6810 (29.7) | 6764 (29.5) |
| Second quartile | 24328 (26.5) | 5909 (25.8) | 6006 (26.2) | 6185 (27.0) | 6228 (27.2) |
| Third quartile | 23040 (25.1) | 5854 (25.5) | 5763 (25.1) | 5641 (24.6) | 5782 (25.2) |
| Fourth quartile | 18159 (19.8) | 5080 (22.2) | 4639 (20.2) | 4289 (18.7) | 4151 (18.1) |
| **BMI, n (%)** |  |  |  |  |  |
| Underweight or normal weight (< 24.9kg/m2) | 36224 (39.5) | 6810 (29.7) | 8387 (36.6) | 9688 (42.3) | 11339 (49.5) |
| Overweight (25.0–29.9kg/m2) | 37740 (41.2) | 9358 (40.8) | 9689 (42.3) | 9582 (41.8) | 9111 (39.7) |
| Obese (≥30kg/m2) | 17736 (19.3) | 6757 (29.5) | 4849 (21.2) | 3655 (15.9) | 2475 (10.8) |
| **Household income before tax per year, (£), n (%)** |  |  |  |  |  |
| Less than 18, 000 | 11986 (13.1) | 4017 (17.5) | 3051 (13.3) | 2582 (11.3) | 2336 (10.2) |
| 18, 000–30, 999 | 19874 (21.7) | 5654 (24.7) | 5106 (22.3) | 4753 (20.7) | 4361 (19.0) |
| 31, 000–51, 999 | 23754 (25.9) | 5458 (23.8) | 5941 (25.9) | 6130 (26.7) | 6225 (27.2) |
| Greater than 52, 000 | 26882 (29.3) | 5199 (22.7) | 6406 (27.9) | 7316 (31.9) | 7961 (34.7) |
| Unknown^a^ | 9204 (10.0) | 2597 (11.3) | 2421 (10.6) | 2144 (9.4) | 2042 (8.9) |
| **Educational group, n (%)** |  |  |  |  |  |
| College or university degree | 39604 (43.2) | 9577 (41.8) | 9776 (42.6) | 9964 (43.5) | 10287 (44.9) |
| Any school degree (A-level, AS-level, O-level, GCSE, CSE) | 34379 (37.5) | 8287 (36.1) | 8576 (37.4) | 8692 (37.9) | 8824 (38.5) |
| Vocational qualification (NVQ, HND, or HNC) or other professional qualifications | 9458 (10.3) | 2565 (11.2) | 2370 (10.3) | 2364 (10.3) | 2159 (9.4) |
| None of the above | 7411 (8.1) | 2238 (9.8) | 1980 (8.6) | 1708 (7.5) | 1485 (6.5) |
| Unknown | 848 (0.9) | 258 (1.1) | 223 (1.0) | 197 (0.9) | 170 (0.7) |
| **Smoking status, n (%)** |  |  |  |  |  |
| Smoker | 6233 (6.8) | 1883 (8.2) | 1562 (6.8) | 1474 (6.4) | 1314 (5.7) |
| Non-smoker | 85467 (93.2) | 21042 (91.8) | 21363 (93.2) | 21451 (93.6) | 21611 (94.3) |
| **Alcohol drinking status, n (%)** |  |  |  |  |  |
| Non-moderate drinking | 30306 (33.0) | 6704 (29.2) | 7456 (32.5) | 7962 (34.7) | 8184 (35.7) |
| Moderate drinking | 61394 (67.0) | 16221 (70.8) | 15469 (67.5) | 14963 (65.3) | 14741 (64.3) |
| **Non-exercisers, n(%)** | 26279 (28.7) | 8636 (37.7) | 7095 (30.9) | 6042 (26.4) | 4506 (19.7) |
| **Exercisers, n (%)** | 65421 (71.3) | 14289 (62.3) | 15830 (69.1) | 16883 (73.6) | 18419 (80.3) |
| **Light activity (min per day), median [IQR]** | 298.21 [255.52, 343.00] | 273.87 [229.65, 321.76] | 295.31 [254.25, 340.99] | 306.77 [265.99, 349.60] | 313.18 [274.15, 354.05] |
| **Moderate activity (min per day), median [IQR]** | 61.92 [42.95, 84.80] | 36.02 [24.82, 49.52] | 55.01 [42.57, 69.74] | 69.99 [55.50, 87.13] | 91.83 [73.01, 114.56] |

**Supplementary Table 6:** Baseline characteristics of participants included in the Parkinson’s disease outcome analysis (n=91, 585) stratified by VILPA standardized frequency.

|  | Overall | Daily VILPA standardized frequency (lasting up to 2 minutes) | | | |
| --- | --- | --- | --- | --- | --- |
|  |  | ≤1.12 | 1.12-2.31 | 2.31-4.39 | >4.39 |
| n | 91585 | 22897 | 22896 | 22896 | 22896 |
| **Age, mean (SD)** | 62.26 (7.85) | 65.14 (7.22) | 62.81 (7.68) | 61.44 (7.73) | 59.63 (7.69) |
| **Sex, n (%)** |  |  |  |  |  |
| Female | 51750 (56.5) | 14620 (63.9) | 13374 (58.4) | 12387 (54.1) | 11369 (49.7) |
| Male | 39835 (43.5) | 8277 (36.1) | 9522 (41.6) | 10509 (45.9) | 11527 (50.3) |
| **Ethnicity, n (%)** |  |  |  |  |  |
| White | 88782 (96.9) | 22297 (97.4) | 22246 (97.2) | 22210 (97.0) | 22029 (96.2) |
| Other | 2803 (3.1) | 600 (2.6) | 650 (2.8) | 686 (3.0) | 867 (3.8) |
| **Socioeconomic status^b^, n (%)** |  |  |  |  |  |
| First quartile | 26126 (28.5) | 6074 (26.5) | 6500 (28.4) | 6801 (29.7) | 6751 (29.5) |
| Second quartile | 24289 (26.5) | 5890 (25.7) | 6004 (26.2) | 6174 (27.0) | 6221 (27.2) |
| Third quartile | 23019 (25.1) | 5853 (25.6) | 5756 (25.1) | 5634 (24.6) | 5776 (25.2) |
| Fourth quartile | 18151 (19.8) | 5080 (22.2) | 4636 (20.2) | 4287 (18.7) | 4148 (18.1) |
| **BMI, n (%)** |  |  |  |  |  |
| Underweight or normal weight (< 24.9kg/m2) | 36174 (39.5) | 6797 (29.7) | 8375 (36.6) | 9680 (42.3) | 11322 (49.4) |
| Overweight (25.0–29.9kg/m2) | 37694 (41.2) | 9342 (40.8) | 9685 (42.3) | 9565 (41.8) | 9102 (39.8) |
| Obese (≥30kg/m2) | 17717 (19.3) | 6758 (29.5) | 4836 (21.1) | 3651 (15.9) | 2472 (10.8) |
| **Household income before tax per year, (£), n (%)** |  |  |  |  |  |
| Less than 18, 000 | 11968 (13.1) | 4007 (17.5) | 3046 (13.3) | 2580 (11.3) | 2335 (10.2) |
| 18, 000–30, 999 | 19860 (21.7) | 5654 (24.7) | 5099 (22.3) | 4757 (20.8) | 4350 (19.0) |
| 31, 000–51, 999 | 23727 (25.9) | 5454 (23.8) | 5935 (25.9) | 6120 (26.7) | 6218 (27.2) |
| Greater than 52, 000 | 26838 (29.3) | 5184 (22.6) | 6402 (28.0) | 7300 (31.9) | 7952 (34.7) |
| Unknown^a^ | 9192 (10.0) | 2598 (11.3) | 2414 (10.5) | 2139 (9.3) | 2041 (8.9) |
| **Educational group, n (%)** |  |  |  |  |  |
| College or university degree | 39542 (43.2) | 9563 (41.8) | 9759 (42.6) | 9945 (43.4) | 10275 (44.9) |
| Any school degree (A-level, AS-level, O-level, GCSE, CSE) | 34330 (37.5) | 8270 (36.1) | 8571 (37.4) | 8679 (37.9) | 8810 (38.5) |
| Vocational qualification (NVQ, HND, or HNC) or other professional qualifications | 9456 (10.3) | 2567 (11.2) | 2366 (10.3) | 2366 (10.3) | 2157 (9.4) |
| None of the above | 7410 (8.1) | 2239 (9.8) | 1978 (8.6) | 1710 (7.5) | 1483 (6.5) |
| Unknown | 847 (0.9) | 258 (1.1) | 222 (1.0) | 196 (0.9) | 171 (0.7) |
| **Smoking status, n (%)** |  |  |  |  |  |
| Smoker | 6229 (6.8) | 1883 (8.2) | 1561 (6.8) | 1474 (6.4) | 1311 (5.7) |
| Non-smoker | 85356 (93.2) | 21014 (91.8) | 21335 (93.2) | 21422 (93.6) | 21585 (94.3) |
| **Alcohol drinking status, n (%)** |  |  |  |  |  |
| Non-moderate drinking | 30270 (33.1) | 6695 (29.2) | 7440 (32.5) | 7962 (34.8) | 8173 (35.7) |
| Moderate drinking | 61315 (66.9) | 16202 (70.8) | 15456 (67.5) | 14934 (65.2) | 14723 (64.3) |
| **Non-exercisers, n(%)** | 26228 (28.6) | 8622 (37.7) | 7085 (30.9) | 6023 (26.3) | 4498 (19.6) |
| **Exercisers, n (%)** | 65357 (71.4) | 14275 (62.3) | 15811 (69.1) | 16873 (73.7) | 18398 (80.4) |
| **Light activity (min per day), median [IQR]** | 298.27 [255.65, 343.05] | 274.27 [230.07, 321.89] | 295.31 [254.26, 341.01] | 306.79 [266.11, 349.67] | 313.11 [274.13, 353.99] |
| **Moderate activity (min per day), median [IQR]** | 61.98 [43.01, 84.85] | 36.15 [24.99, 49.61] | 55.10 [42.61, 69.82] | 70.07 [55.56, 87.18] | 91.85 [73.02, 114.56] |

**Supplementary Table 7:** Baseline characteristics of participants included in the stroke outcome analysis (n=89, 405) stratified by VILPA standardized frequency.

|  | Overall | Daily VILPA standardized frequency (lasting up to 2 minutes) | | | |
| --- | --- | --- | --- | --- | --- |
|  |  | ≤1.13 | 1.13-2.33 | 2.33-4.43 | >4.43 |
| n | 89405 | 22352 | 22351 | 22351 | 22351 |
| **Age, mean (SD)** | 62.15 (7.84) | 65.01 (7.24) | 62.69 (7.68) | 61.33 (7.74) | 59.56 (7.67) |
| **Sex, n (%)** |  |  |  |  |  |
| Female | 50831 (56.9) | 14384 (64.4) | 13154 (58.9) | 12131 (54.3) | 11162 (49.9) |
| Male | 38574 (43.1) | 7968 (35.6) | 9197 (41.1) | 10220 (45.7) | 11189 (50.1) |
| **Ethnicity, n (%)** |  |  |  |  |  |
| White | 86636 (96.9) | 21756 (97.3) | 21713 (97.1) | 21670 (97.0) | 21497 (96.2) |
| Other | 2769 (3.1) | 596 (2.7) | 638 (2.9) | 681 (3.0) | 854 (3.8) |
| **Socioeconomic status^b^, n (%)** |  |  |  |  |  |
| First quartile | 25515 (28.5) | 5947 (26.6) | 6368 (28.5) | 6613 (29.6) | 6587 (29.5) |
| Second quartile | 23728 (26.5) | 5759 (25.8) | 5858 (26.2) | 6039 (27.0) | 6072 (27.2) |
| Third quartile | 22484 (25.1) | 5705 (25.5) | 5630 (25.2) | 5511 (24.7) | 5638 (25.2) |
| Fourth quartile | 17678 (19.8) | 4941 (22.1) | 4495 (20.1) | 4188 (18.7) | 4054 (18.1) |
| **BMI, n (%)** |  |  |  |  |  |
| Underweight or normal weight (< 24.9kg/m2) | 35520 (39.7) | 6722 (30.1) | 8222 (36.8) | 9474 (42.4) | 11102 (49.7) |
| Overweight (25.0–29.9kg/m2) | 36763 (41.1) | 9127 (40.8) | 9431 (42.2) | 9342 (41.8) | 8863 (39.7) |
| Obese (≥30kg/m2) | 17122 (19.2) | 6503 (29.1) | 4698 (21.0) | 3535 (15.8) | 2386 (10.7) |
| **Household income before tax per year, (£), n (%)** |  |  |  |  |  |
| Less than 18, 000 | 11512 (12.9) | 3836 (17.2) | 2911 (13.0) | 2496 (11.2) | 2269 (10.2) |
| 18, 000–30, 999 | 19274 (21.6) | 5475 (24.5) | 4969 (22.2) | 4603 (20.6) | 4227 (18.9) |
| 31, 000–51, 999 | 23233 (26.0) | 5349 (23.9) | 5810 (26.0) | 5982 (26.8) | 6092 (27.3) |
| Greater than 52, 000 | 26434 (29.6) | 5151 (23.0) | 6323 (28.3) | 7180 (32.1) | 7780 (34.8) |
| Unknown^a^ | 8952 (10.0) | 2541 (11.4) | 2338 (10.5) | 2090 (9.4) | 1983 (8.9) |
| **Educational group, n (%)** |  |  |  |  |  |
| College or university degree | 38779 (43.4) | 9413 (42.1) | 9587 (42.9) | 9758 (43.7) | 10021 (44.8) |
| Any school degree (A-level, AS-level, O-level, GCSE, CSE) | 33524 (37.5) | 8061 (36.1) | 8359 (37.4) | 8477 (37.9) | 8627 (38.6) |
| Vocational qualification (NVQ, HND, or HNC) or other professional qualifications | 9144 (10.2) | 2479 (11.1) | 2283 (10.2) | 2284 (10.2) | 2098 (9.4) |
| None of the above | 7137 (8.0) | 2146 (9.6) | 1908 (8.5) | 1643 (7.4) | 1440 (6.4) |
| Unknown | 821 (0.9) | 253 (1.1) | 214 (1.0) | 189 (0.8) | 165 (0.7) |
| **Smoking status, n (%)** |  |  |  |  |  |
| Smoker | 6052 (6.8) | 1822 (8.2) | 1512 (6.8) | 1436 (6.4) | 1282 (5.7) |
| Non-smoker | 83353 (93.2) | 20530 (91.8) | 20839 (93.2) | 20915 (93.6) | 21069 (94.3) |
| **Alcohol drinking status, n (%)** |  |  |  |  |  |
| Non-moderate drinking | 29491 (33.0) | 6523 (29.2) | 7242 (32.4) | 7762 (34.7) | 7964 (35.6) |
| Moderate drinking | 59914 (67.0) | 15829 (70.8) | 15109 (67.6) | 14589 (65.3) | 14387 (64.4) |
| **Non-exercisers, n(%)** | 25435 (28.4) | 8344 (37.3) | 6879 (30.8) | 5848 (26.2) | 4364 (19.5) |
| **Exercisers, n (%)** | 63970 (71.6) | 14008 (62.7) | 15472 (69.2) | 16503 (73.8) | 17987 (80.5) |
| **Light activity (min per day), median [IQR]** | 298.58 [256.04, 343.24] | 274.96 [230.67, 322.31] | 295.86 [254.75, 341.36] | 306.88 [266.19, 349.67] | 313.25 [274.31, 354.36] |
| **Moderate activity (min per day), median [IQR]** | 62.29 [43.32, 85.14] | 36.50 [25.31, 50.04] | 55.40 [42.92, 70.07] | 70.45 [55.80, 87.46] | 92.20 [73.31, 114.95] |

**Supplementary Table 8:** Comparison of cumulative incidence of brain diorders across VILPA frequency and duration quartiles (1min/2min) with log-rank test results.

| Outcome | Exposure | Comparison | P_value |
| --- | --- | --- | --- |
| Anxiety | VILPA Freq (1min) | Log-rank | 0 |
|  |  | Q2 vs Q1 | 1.59E-09 |
|  |  | Q3 vs Q1 | 3.54E-10 |
|  |  | Q4 vs Q1 | 0 |
|  | VILPA Dur (1min) | Log-rank | 0 |
|  |  | Q2 vs Q1 | 1.46E-09 |
|  |  | Q3 vs Q1 | 3.35E-11 |
|  |  | Q4 vs Q1 | 0 |
|  | VILPA Freq (2min) | Log-rank | 0 |
|  |  | Q2 vs Q1 | 3.65E-09 |
|  |  | Q3 vs Q1 | 1.70E-11 |
|  |  | Q4 vs Q1 | 0 |
|  | VILPA Dur (2min) | Log-rank | 0 |
|  |  | Q2 vs Q1 | 2.79E-08 |
|  |  | Q3 vs Q1 | 1.10E-11 |
|  |  | Q4 vs Q1 | 0 |
| Depression | VILPA Freq (1min) | Log-rank | 0 |
|  |  | Q2 vs Q1 | 1.74E-06 |
|  |  | Q3 vs Q1 | 1.18E-09 |
|  |  | Q4 vs Q1 | 1.11E-16 |
|  | VILPA Dur (1min) | Log-rank | 1.11E-16 |
|  |  | Q2 vs Q1 | 1.47E-05 |
|  |  | Q3 vs Q1 | 3.05E-09 |
|  |  | Q4 vs Q1 | 1.11E-16 |
|  | VILPA Freq (2min) | Log-rank | 4.44E-16 |
|  |  | Q2 vs Q1 | 8.90E-06 |
|  |  | Q3 vs Q1 | 5.71E-09 |
|  |  | Q4 vs Q1 | 4.44E-16 |
|  | VILPA Dur (2min) | Log-rank | 6.66E-16 |
|  |  | Q2 vs Q1 | 4.32E-06 |
|  |  | Q3 vs Q1 | 1.04E-08 |
|  |  | Q4 vs Q1 | 5.55E-16 |
| Dementia | VILPA Freq (1min) | Log-rank | 0 |
|  |  | Q2 vs Q1 | 2.55E-15 |
|  |  | Q3 vs Q1 | 0.00E+00 |
|  |  | Q4 vs Q1 | 0 |
|  | VILPA Dur (1min) | Log-rank | 0 |
|  |  | Q2 vs Q1 | 1.55E-15 |
|  |  | Q3 vs Q1 | 0.00E+00 |
|  |  | Q4 vs Q1 | 0 |
|  | VILPA Freq (2min) | Log-rank | 0 |
|  |  | Q2 vs Q1 | 4.44E-16 |
|  |  | Q3 vs Q1 | 0.00E+00 |
|  |  | Q4 vs Q1 | 0 |
|  | VILPA Dur (2min) | Log-rank | 0 |
|  |  | Q2 vs Q1 | 0.00E+00 |
|  |  | Q3 vs Q1 | 0.00E+00 |
|  |  | Q4 vs Q1 | 0 |
| Parkinson’s disease | VILPA Freq (1min) | Log-rank | 0 |
|  |  | Q2 vs Q1 | 1.11E-16 |
|  |  | Q3 vs Q1 | 0.00E+00 |
|  |  | Q4 vs Q1 | 0 |
|  | VILPA Dur (1min) | Log-rank | 0 |
|  |  | Q2 vs Q1 | 1.11E-16 |
|  |  | Q3 vs Q1 | 0.00E+00 |
|  |  | Q4 vs Q1 | 0 |
|  | VILPA Freq (2min) | Log-rank | 0 |
|  |  | Q2 vs Q1 | 3.33E-16 |
|  |  | Q3 vs Q1 | 0.00E+00 |
|  |  | Q4 vs Q1 | 0 |
|  | VILPA Dur (2min) | Log-rank | 0 |
|  |  | Q2 vs Q1 | 0.00E+00 |
|  |  | Q3 vs Q1 | 0.00E+00 |
|  |  | Q4 vs Q1 | 0 |
| Stroke | VILPA Freq (1min) | Log-rank | 0 |
|  |  | Q2 vs Q1 | 5.20E-06 |
|  |  | Q3 vs Q1 | 2.62E-09 |
|  |  | Q4 vs Q1 | 0 |
|  | VILPA Dur (1min) | Log-rank | 0 |
|  |  | Q2 vs Q1 | 2.94E-06 |
|  |  | Q3 vs Q1 | 5.06E-09 |
|  |  | Q4 vs Q1 | 0 |
|  | VILPA Freq (2min) | Log-rank | 0 |
|  |  | Q2 vs Q1 | 1.24E-05 |
|  |  | Q3 vs Q1 | 1.51E-09 |
|  |  | Q4 vs Q1 | 0 |
|  | VILPA Dur (2min) | Log-rank | 0 |
|  |  | Q2 vs Q1 | 1.62E-06 |
|  |  | Q3 vs Q1 | 4.41E-10 |
|  |  | Q4 vs Q1 | 0 |

**Supplementary Figure 1:** Dose-response associations between four VILPA metrics and brain disorders.

VILPA: vigorous intermittent lifestyle physical activity

Model was adjusted for age, sex, ethnicity, socioeconomic status, BMI, household income before tax per year, educational group, smoking status, alcohol drinking status, duration of light physical activity, duration of moderate physical activity and vigorous physical activity lasting more than one/two minute. Shaded region with dashed lines represents 95%CI. Solid line represents point estimate.

**Supplementary Table 9:** Moderation analyses of the associations between VILPA and brain disorders by exercise status

| Outcome | Exposure | P for Interaction |
| --- | --- | --- |
| Anxiety | VILPA Freq (1min) | 0.5296 |
|  | VILPA Dur (1min) | 0.501363 |
|  | VILPA Freq (2min) | 0.488369 |
|  | VILPA Dur (2min) | 0.431499 |
| Depression | VILPA Freq (1min) | 0.210016 |
|  | VILPA Dur (1min) | 0.196038 |
|  | VILPA Freq (2min) | 0.217886 |
|  | VILPA Dur (2min) | 0.207601 |
| Dementia | VILPA Freq (1min) | 0.241584 |
|  | VILPA Dur (1min) | 0.267795 |
|  | VILPA Freq (2min) | 0.286705 |
|  | VILPA Dur (2min) | 0.370013 |
| Parkinson's disease | VILPA Freq (1min) | 0.6334 |
|  | VILPA Dur (1min) | 0.599704 |
|  | VILPA Freq (2min) | 0.608068 |
|  | VILPA Dur (2min) | 0.564625 |
| Stroke | VILPA Freq (1min) | 0.371205 |
|  | VILPA Dur (1min) | 0.363502 |
|  | VILPA Freq (2min) | 0.344706 |
|  | VILPA Dur (2min) | 0.342295 |

**Supplementary Table 10:** E-values for dementia, stroke, Parkinson’s disease, depressive disorders, and anxiety disorders

| Outcome | Bouts | VILPA duration | | VILPA frequency | |
| --- | --- | --- | --- | --- | --- |
|  |  | E | CI | E | CI |
| Anxiety | last up to 1min |  |  |  |  |
|  | Q1 | ref | - | - | - |
|  | Q2 | 1.84 | 1.5 | 1.83 | 1.48 |
|  | Q3 | 1.7 | 1.29 | 1.62 | 1.19 |
|  | Q4 | 2.06 | 1.57 | 1.98 | 1.5 |
|  | last up to 2min |  |  |  |  |
|  | Q1 | ref | - | - | - |
|  | Q2 | 1.73 | 1.38 | 1.81 | 1.46 |
|  | Q3 | 1.68 | 1.27 | 1.71 | 1.31 |
|  | Q4 | 1.87 | 1.38 | 2.01 | 1.53 |
| Depression | last up to 1min |  |  |  |  |
|  | Q1 | ref | - | - | - |
|  | Q2 | 1.57 | 1.05 | 1.67 | 1.22 |
|  | Q3 | 1.6 | 1 | 1.64 | 1.08 |
|  | Q4 | 1.8 | 1.18 | 1.79 | 1.17 |
|  | last up to 2min |  |  |  |  |
|  | Q1 | ref | - | - | - |
|  | Q2 | 1.6 | 1.12 | 1.58 | 1.08 |
|  | Q3 | 1.51 | 1 | 1.55 | 1 |
|  | Q4 | 1.68 | 1 | 1.72 | 1 |
| Dementia | last up to 1min |  |  |  |  |
|  | Q1 | ref | - | - | - |
|  | Q2 | 2.08 | 1.49 | 2.07 | 1.48 |
|  | Q3 | 2.46 | 1.69 | 2.61 | 1.81 |
|  | Q4 | 2.6 | 1.57 | 2.59 | 1.56 |
|  | last up to 2min |  |  |  |  |
|  | Q1 | ref | - | - | - |
|  | Q2 | 2.25 | 1.64 | 2.14 | 1.54 |
|  | Q3 | 2.47 | 1.7 | 2.32 | 1.57 |
|  | Q4 | 2.56 | 1.55 | 2.6 | 1.58 |
| Parkinson’s disease | last up to 1min |  |  |  |  |
|  | Q1 | ref | - | - | - |
|  | Q2 | 2.93 | 1.96 | 2.92 | 1.94 |
|  | Q3 | 2.88 | 1.71 | 2.82 | 1.66 |
|  | Q4 | 2.65 | 1.14 | 2.57 | 1 |
|  | last up to 2min |  |  |  |  |
|  | Q1 | ref | - | - | - |
|  | Q2 | 3.03 | 2.02 | 2.84 | 1.88 |
|  | Q3 | 2.56 | 1.47 | 2.81 | 1.65 |
|  | Q4 | 2.76 | 1.26 | 2.56 | 1 |
| Stroke | last up to 1min |  |  |  |  |
|  | Q1 | ref | - | - | - |
|  | Q2 | 1.54 | 1 | 1.52 | 1 |
|  | Q3 | 1.45 | 1 | 1.49 | 1 |
|  | Q4 | 1.79 | 1.12 | 1.86 | 1.23 |
|  | last up to 2min |  |  |  |  |
|  | Q1 | ref | - | - | - |
|  | Q2 | 1.59 | 1.05 | 1.48 | 1 |
|  | Q3 | 1.57 | 1 | 1.51 | 1 |
|  | Q4 | 1.89 | 1.27 | 1.83 | 1.19 |

E-values estimate the minimum strength of association on the risk ratio scale that a potential confounder would need to have with the exposure and outcome to nullify the observed association. E denotes E-values for the point estimate and CI denotes the E-value for the confidence interval bound closest to the null. E-values were calculated based on the fully adjusted estimates from Model 2.

**Supplementary Table 11:** Additional adjustments for self-rated health status

| Outcome | Group | Term | HR | L95 | U95 | P |
| --- | --- | --- | --- | --- | --- | --- |
| Anxiety | Frequency (≤1min) | Q2 | 0.79 | 0.71 | 0.89 | 0.0002 |
|  |  | Q3 | 0.86 | 0.75 | 0.98 | 0.0211 |
|  |  | Q4 | 0.76 | 0.64 | 0.89 | 0.0009 |
|  | Duration (≤1min) | Q2 | 0.79 | 0.7 | 0.89 | 0.0001 |
|  |  | Q3 | 0.83 | 0.73 | 0.95 | 0.0066 |
|  |  | Q4 | 0.74 | 0.63 | 0.87 | 0.0003 |
|  | Frequency (≤2min) | Q2 | 0.8 | 0.71 | 0.9 | 0.0003 |
|  |  | Q3 | 0.83 | 0.73 | 0.95 | 0.0056 |
|  |  | Q4 | 0.75 | 0.64 | 0.88 | 0.0005 |
|  | Duration (≤2min) | Q2 | 0.82 | 0.73 | 0.93 | 0.0012 |
|  |  | Q3 | 0.84 | 0.73 | 0.96 | 0.0089 |
|  |  | Q4 | 0.79 | 0.67 | 0.92 | 0.0036 |
| Depression | Frequency (≤1min) | Q2 | 0.84 | 0.73 | 0.97 | 0.0163 |
|  |  | Q3 | 0.85 | 0.73 | 1 | 0.0455 |
|  |  | Q4 | 0.81 | 0.66 | 0.98 | 0.0314 |
|  | Duration (≤1min) | Q2 | 0.87 | 0.76 | 1 | 0.0499 |
|  |  | Q3 | 0.86 | 0.73 | 1.01 | 0.0635 |
|  |  | Q4 | 0.8 | 0.66 | 0.98 | 0.0297 |
|  | Frequency (≤2min) | Q2 | 0.87 | 0.75 | 1 | 0.0448 |
|  |  | Q3 | 0.88 | 0.75 | 1.03 | 0.1 |
|  |  | Q4 | 0.83 | 0.68 | 1.01 | 0.0569 |
|  | Duration (≤2min) | Q2 | 0.86 | 0.75 | 0.99 | 0.0364 |
|  |  | Q3 | 0.89 | 0.76 | 1.04 | 0.1458 |
|  |  | Q4 | 0.84 | 0.69 | 1.02 | 0.0744 |
| Dementia | Frequency (≤1min) | Q2 | 0.73 | 0.6 | 0.9 | 0.0024 |
|  |  | Q3 | 0.62 | 0.48 | 0.8 | 0.0002 |
|  |  | Q4 | 0.62 | 0.45 | 0.87 | 0.0055 |
|  | Duration (≤1min) | Q2 | 0.73 | 0.6 | 0.89 | 0.0022 |
|  |  | Q3 | 0.65 | 0.5 | 0.83 | 0.0007 |
|  |  | Q4 | 0.62 | 0.45 | 0.87 | 0.0052 |
|  | Frequency (≤2min) | Q2 | 0.72 | 0.59 | 0.88 | 0.0012 |
|  |  | Q3 | 0.68 | 0.53 | 0.87 | 0.0021 |
|  |  | Q4 | 0.62 | 0.44 | 0.87 | 0.0051 |
|  | Duration (≤2min) | Q2 | 0.69 | 0.57 | 0.85 | 0.0004 |
|  |  | Q3 | 0.65 | 0.5 | 0.83 | 0.0006 |
|  |  | Q4 | 0.63 | 0.45 | 0.87 | 0.0058 |
| Parkinson’s disease | Frequency (≤1min) | Q2 | 0.57 | 0.42 | 0.76 | 0.0002 |
|  |  | Q3 | 0.58 | 0.4 | 0.84 | 0.004 |
|  |  | Q4 | 0.63 | 0.39 | 1.01 | 0.0531 |
|  | Duration (≤1min) | Q2 | 0.56 | 0.42 | 0.76 | 0.0002 |
|  |  | Q3 | 0.57 | 0.4 | 0.83 | 0.0029 |
|  |  | Q4 | 0.61 | 0.38 | 0.98 | 0.0424 |
|  | Frequency (≤2min) | Q2 | 0.58 | 0.43 | 0.78 | 0.0003 |
|  |  | Q3 | 0.58 | 0.41 | 0.84 | 0.0041 |
|  |  | Q4 | 0.63 | 0.39 | 1.01 | 0.0541 |
|  | Duration (≤2min) | Q2 | 0.55 | 0.41 | 0.74 | 0.0001 |
|  |  | Q3 | 0.63 | 0.44 | 0.9 | 0.0108 |
|  |  | Q4 | 0.59 | 0.37 | 0.96 | 0.032 |
| Stroke | Frequency (≤1min) | Q2 | 0.88 | 0.76 | 1.02 | 0.0922 |
|  |  | Q3 | 0.89 | 0.76 | 1.05 | 0.1672 |
|  |  | Q4 | 0.79 | 0.64 | 0.96 | 0.0212 |
|  | Duration (≤1min) | Q2 | 0.88 | 0.76 | 1.01 | 0.0774 |
|  |  | Q3 | 0.9 | 0.77 | 1.06 | 0.2196 |
|  |  | Q4 | 0.81 | 0.66 | 0.99 | 0.038 |
|  | Frequency (≤2min) | Q2 | 0.89 | 0.77 | 1.03 | 0.129 |
|  |  | Q3 | 0.88 | 0.75 | 1.04 | 0.1448 |
|  |  | Q4 | 0.79 | 0.65 | 0.97 | 0.0268 |
|  | Duration (≤2min) | Q2 | 0.86 | 0.75 | 1 | 0.0457 |
|  |  | Q3 | 0.87 | 0.74 | 1.02 | 0.0943 |
|  |  | Q4 | 0.78 | 0.63 | 0.95 | 0.0159 |

**Supplementary Table 12:** Additional adjustments for cardiovascular disease outcomes

| Outcome | Group | Term | HR | L95 | U95 | P |
| --- | --- | --- | --- | --- | --- | --- |
| Anxiety | Frequency (≤1min) | Q2 | 0.8 | 0.71 | 0.91 | 0.0003 |
|  |  | Q3 | 0.87 | 0.76 | 0.99 | 0.0408 |
|  |  | Q4 | 0.77 | 0.65 | 0.91 | 0.0021 |
|  | Duration (≤1min) | Q2 | 0.8 | 0.71 | 0.9 | 0.0003 |
|  |  | Q3 | 0.85 | 0.74 | 0.97 | 0.014 |
|  |  | Q4 | 0.75 | 0.64 | 0.89 | 0.0007 |
|  | Frequency (≤2min) | Q2 | 0.81 | 0.72 | 0.91 | 0.0006 |
|  |  | Q3 | 0.84 | 0.74 | 0.96 | 0.012 |
|  |  | Q4 | 0.76 | 0.65 | 0.9 | 0.0014 |
|  | Duration (≤2min) | Q2 | 0.83 | 0.74 | 0.94 | 0.0025 |
|  |  | Q3 | 0.85 | 0.75 | 0.97 | 0.0192 |
|  |  | Q4 | 0.8 | 0.68 | 0.94 | 0.0082 |
| Depression | Frequency (≤1min) | Q2 | 0.86 | 0.75 | 0.99 | 0.0356 |
|  |  | Q3 | 0.87 | 0.75 | 1.03 | 0.0989 |
|  |  | Q4 | 0.83 | 0.68 | 1.01 | 0.0675 |
|  | Duration (≤1min) | Q2 | 0.89 | 0.77 | 1.02 | 0.0987 |
|  |  | Q3 | 0.88 | 0.75 | 1.04 | 0.1315 |
|  |  | Q4 | 0.83 | 0.68 | 1.01 | 0.0652 |
|  | Frequency (≤2min) | Q2 | 0.88 | 0.77 | 1.02 | 0.0884 |
|  |  | Q3 | 0.9 | 0.77 | 1.05 | 0.193 |
|  |  | Q4 | 0.85 | 0.7 | 1.04 | 0.1132 |
|  | Duration (≤2min) | Q2 | 0.88 | 0.76 | 1.01 | 0.0715 |
|  |  | Q3 | 0.91 | 0.78 | 1.07 | 0.2698 |
|  |  | Q4 | 0.86 | 0.71 | 1.05 | 0.1441 |
| Dementia | Frequency (≤1min) | Q2 | 0.75 | 0.61 | 0.91 | 0.0044 |
|  |  | Q3 | 0.64 | 0.49 | 0.82 | 0.0006 |
|  |  | Q4 | 0.64 | 0.46 | 0.9 | 0.0097 |
|  | Duration (≤1min) | Q2 | 0.75 | 0.61 | 0.91 | 0.0041 |
|  |  | Q3 | 0.67 | 0.52 | 0.86 | 0.0018 |
|  |  | Q4 | 0.64 | 0.46 | 0.9 | 0.0094 |
|  | Frequency (≤2min) | Q2 | 0.73 | 0.6 | 0.9 | 0.0025 |
|  |  | Q3 | 0.7 | 0.54 | 0.9 | 0.0048 |
|  |  | Q4 | 0.64 | 0.46 | 0.9 | 0.0093 |
|  | Duration (≤2min) | Q2 | 0.7 | 0.58 | 0.86 | 0.0007 |
|  |  | Q3 | 0.67 | 0.52 | 0.86 | 0.0016 |
|  |  | Q4 | 0.65 | 0.47 | 0.9 | 0.0105 |
| Parkinson’s disease | Frequency (≤1min) | Q2 | 0.56 | 0.42 | 0.76 | 0.0002 |
|  |  | Q3 | 0.58 | 0.4 | 0.83 | 0.0033 |
|  |  | Q4 | 0.62 | 0.38 | 0.99 | 0.0461 |
|  | Duration (≤1min) | Q2 | 0.56 | 0.42 | 0.75 | 0.0001 |
|  |  | Q3 | 0.57 | 0.39 | 0.82 | 0.0024 |
|  |  | Q4 | 0.6 | 0.38 | 0.97 | 0.0367 |
|  | Frequency (≤2min) | Q2 | 0.58 | 0.43 | 0.77 | 0.0002 |
|  |  | Q3 | 0.58 | 0.4 | 0.83 | 0.0034 |
|  |  | Q4 | 0.62 | 0.39 | 0.99 | 0.047 |
|  | Duration (≤2min) | Q2 | 0.55 | 0.4 | 0.74 | 0.0001 |
|  |  | Q3 | 0.62 | 0.43 | 0.89 | 0.0089 |
|  |  | Q4 | 0.58 | 0.36 | 0.94 | 0.0276 |
| Stroke | Frequency (≤1min) | Q2 | 0.9 | 0.78 | 1.04 | 0.152 |
|  |  | Q3 | 0.92 | 0.78 | 1.08 | 0.2957 |
|  |  | Q4 | 0.81 | 0.66 | 1 | 0.0485 |
|  | Duration (≤1min) | Q2 | 0.89 | 0.77 | 1.03 | 0.1329 |
|  |  | Q3 | 0.93 | 0.79 | 1.09 | 0.3703 |
|  |  | Q4 | 0.83 | 0.68 | 1.02 | 0.0822 |
|  | Frequency (≤2min) | Q2 | 0.91 | 0.79 | 1.05 | 0.2097 |
|  |  | Q3 | 0.91 | 0.77 | 1.07 | 0.2578 |
|  |  | Q4 | 0.82 | 0.67 | 1.01 | 0.0598 |
|  | Duration (≤2min) | Q2 | 0.88 | 0.76 | 1.02 | 0.0791 |
|  |  | Q3 | 0.89 | 0.76 | 1.05 | 0.1783 |
|  |  | Q4 | 0.8 | 0.66 | 0.99 | 0.0369 |

**Supplementary Table 13:** Additional adjustments for cognitive function

| Outcome | Group | Term | HR | L95 | U95 | P |
| --- | --- | --- | --- | --- | --- | --- |
| Anxiety | Frequency (≤1min) | Q2 | 0.8 | 0.71 | 0.9 | 0.0002 |
|  |  | Q3 | 0.86 | 0.75 | 0.98 | 0.0226 |
|  |  | Q4 | 0.76 | 0.64 | 0.89 | 0.001 |
|  | Duration (≤1min) | Q2 | 0.79 | 0.7 | 0.89 | 0.0001 |
|  |  | Q3 | 0.83 | 0.73 | 0.95 | 0.007 |
|  |  | Q4 | 0.74 | 0.63 | 0.87 | 0.0003 |
|  | Frequency (≤2min) | Q2 | 0.8 | 0.71 | 0.9 | 0.0003 |
|  |  | Q3 | 0.83 | 0.73 | 0.95 | 0.006 |
|  |  | Q4 | 0.75 | 0.64 | 0.88 | 0.0006 |
|  | Duration (≤2min) | Q2 | 0.82 | 0.73 | 0.93 | 0.0014 |
|  |  | Q3 | 0.84 | 0.74 | 0.96 | 0.0096 |
|  |  | Q4 | 0.79 | 0.67 | 0.93 | 0.004 |
| Depression | Frequency (≤1min) | Q2 | 0.84 | 0.73 | 0.97 | 0.0171 |
|  |  | Q3 | 0.85 | 0.73 | 1 | 0.0457 |
|  |  | Q4 | 0.81 | 0.66 | 0.98 | 0.0332 |
|  | Duration (≤1min) | Q2 | 0.87 | 0.76 | 1 | 0.0514 |
|  |  | Q3 | 0.86 | 0.73 | 1.01 | 0.0635 |
|  |  | Q4 | 0.81 | 0.66 | 0.98 | 0.0313 |
|  | Frequency (≤2min) | Q2 | 0.87 | 0.75 | 1 | 0.0467 |
|  |  | Q3 | 0.88 | 0.75 | 1.03 | 0.1 |
|  |  | Q4 | 0.83 | 0.68 | 1.01 | 0.0595 |
|  | Duration (≤2min) | Q2 | 0.86 | 0.75 | 0.99 | 0.0378 |
|  |  | Q3 | 0.89 | 0.76 | 1.04 | 0.1474 |
|  |  | Q4 | 0.84 | 0.69 | 1.02 | 0.0773 |
| Dementia | Frequency (≤1min) | Q2 | 0.73 | 0.6 | 0.9 | 0.0024 |
|  |  | Q3 | 0.62 | 0.48 | 0.8 | 0.0002 |
|  |  | Q4 | 0.62 | 0.45 | 0.87 | 0.0055 |
|  | Duration (≤1min) | Q2 | 0.73 | 0.6 | 0.89 | 0.0022 |
|  |  | Q3 | 0.65 | 0.5 | 0.83 | 0.0007 |
|  |  | Q4 | 0.62 | 0.45 | 0.87 | 0.0052 |
|  | Frequency (≤2min) | Q2 | 0.72 | 0.59 | 0.88 | 0.0013 |
|  |  | Q3 | 0.68 | 0.53 | 0.87 | 0.0021 |
|  |  | Q4 | 0.62 | 0.44 | 0.87 | 0.0051 |
|  | Duration (≤2min) | Q2 | 0.69 | 0.57 | 0.85 | 0.0004 |
|  |  | Q3 | 0.65 | 0.5 | 0.83 | 0.0006 |
|  |  | Q4 | 0.63 | 0.45 | 0.87 | 0.0058 |
| Parkinson’s disease | Frequency (≤1min) | Q2 | 0.57 | 0.42 | 0.77 | 0.0002 |
|  |  | Q3 | 0.58 | 0.41 | 0.84 | 0.0042 |
|  |  | Q4 | 0.63 | 0.39 | 1.01 | 0.0548 |
|  | Duration (≤1min) | Q2 | 0.57 | 0.42 | 0.76 | 0.0002 |
|  |  | Q3 | 0.57 | 0.4 | 0.83 | 0.0031 |
|  |  | Q4 | 0.61 | 0.38 | 0.99 | 0.0438 |
|  | Frequency (≤2min) | Q2 | 0.58 | 0.43 | 0.78 | 0.0003 |
|  |  | Q3 | 0.59 | 0.41 | 0.85 | 0.0043 |
|  |  | Q4 | 0.63 | 0.39 | 1.01 | 0.0558 |
|  | Duration (≤2min) | Q2 | 0.55 | 0.41 | 0.75 | 0.0001 |
|  |  | Q3 | 0.63 | 0.44 | 0.9 | 0.0112 |
|  |  | Q4 | 0.59 | 0.37 | 0.96 | 0.0328 |
| Stroke | Frequency (≤1min) | Q2 | 0.88 | 0.76 | 1.02 | 0.0923 |
|  |  | Q3 | 0.89 | 0.76 | 1.05 | 0.167 |
|  |  | Q4 | 0.79 | 0.64 | 0.96 | 0.0212 |
|  | Duration (≤1min) | Q2 | 0.88 | 0.76 | 1.01 | 0.0775 |
|  |  | Q3 | 0.9 | 0.77 | 1.06 | 0.2195 |
|  |  | Q4 | 0.81 | 0.66 | 0.99 | 0.0381 |
|  | Frequency (≤2min) | Q2 | 0.89 | 0.77 | 1.03 | 0.129 |
|  |  | Q3 | 0.88 | 0.75 | 1.04 | 0.1446 |
|  |  | Q4 | 0.79 | 0.65 | 0.97 | 0.0268 |
|  | Duration (≤2min) | Q2 | 0.86 | 0.75 | 1 | 0.0457 |
|  |  | Q3 | 0.87 | 0.74 | 1.02 | 0.0941 |
|  |  | Q4 | 0.78 | 0.63 | 0.95 | 0.0159 |
